# Effects of husbandry systems and Chinese indigenous chicken strain on cecum microbial diversity

**DOI:** 10.5713/ajas.19.0157

**Published:** 2019-10-21

**Authors:** Xiuxue Dong, Bing Hu, Wenlong Wan, Yanzhang Gong, Yanping Feng

**Affiliations:** 1 College of Animal Science and Veterinary Medicine, Huazhong Agricultural University, Wuhan, Hubei 430070, China

**Keywords:** Jingyang Chicken, Husbandry System, Cecum Microbiome, Diversity, Illumina MiSeq Sequencing

## Abstract

**Objective:**

This study was to evaluate the effect of husbandry systems and strains on cecum microbial diversity of Jingyang chickens under the same dietary conditions.

**Methods:**

A total of 320 laying hens (body weight, 1.70±0.15 kg; 47 weeks old) were randomly allocated to one of the four treatments: i) Silver-feathered hens in enrichment cages (SEC) with an individual cage (70×60×75 cm), ii) Silver-feathered hens in free range (SFR) with the stocking density of 1.5 chickens per ten square meters, iii) Gold-feathered hens in enrichment cages (GEC), iv) Gold-feathered hens in free range (GFR). The experiment lasted 8 weeks and the cecum fecal samples were collected for 16S rDNA high throughput sequencing at the end of experiment.

**Results:**

i) The core microbiota was composed of Bacteroidetes (49% to 60%), Firmicutes (21% to 32%) and Proteobacteria (2% to 4%) at the phylum level. ii) The core bacteria were Bacteroides (26% to 31%), Rikenellaceae (9% to 16%), Parabacteroides (2% to 5%) and Lachnoclostridium (2% to 6%) at the genus level. iii) The indexes of operational taxonomic unit, Shannon, Simpson and observed species were all higher in SFR group than in SEC group while in GEC group than in GFR group, with SFR group showing the greatest diversity of cecum microorganisms among the four groups. iv) The clustering result was consistent with the strain classification, with a similar composition of cecum bacteria in the two strains of laying hens.

**Conclusion:**

The core microbiota were not altered by husbandry systems or strains. The free-range system increased the diversity of cecal microbes only for silver feathered hens. However, the cecum microbial composition was similar in two strain treatments under the same dietary conditions.

## INTRODUCTION

Asian indigenous chickens under the traditional husbandry system (free range) enjoy the advantages of better adaptation to endemic diseases and other harsh environmental stresses as well as requiring minimal care and low cost, which are in accordance with the wishes of local farmers [1]. The meat and eggs of some indigenous chickens are preferred over those of exotic breeds because of their unique flavor; meanwhile, the international voice for animal welfare allows the chicken industry to become more competitive by stressing the welfare advantages of the free-range system. This system is not only environmentally friendly and enables the animals to enjoy higher welfare standards and produce agricultural products with better quality and better flavor [2], but also provides job opportunities to the low-income population in rural areas [3].

Jingyang chicken, a Chinese indigenous breed from Jianshi county, Hubei, China with a unique appearance and good meat quality, has two strains of silver feather and gold feather. The quality and flavor of chicken products are related to the intestinal microbial community, which not only provides nutrition for the host, but also helps to maintain the balance of the intestinal microecology, and ensures the health and development of the intestinal tract [4, 5]. Particularly, the core flora of intestinal tract directly affects its normal function [6], which is closely related to the chicken products such as meat and eggs. Moreover, intestinal microbial community can be jointly affected by genetics and husbandry systems. The gut microflora is a complex ecosystem with a symbiotic relationship to the host, and interactions between them affect the physiological, immune and nutritional status of the host [7]. In general, the intestinal microbial system mainly focuses on microbial community composition and diversity. The intestinal microbial diversity is essential for animals to digest and absorb nutrients, maintain intestinal biochemical and physiological functions, and promote the development of immune system [8,9].

Unlike ruminants which mainly depend on ruminal microorganisms to digest cellulose, monogastric animals such as chickens are primarily dependent on the fermentation and degradation of microbes in the large intestine for digestion of cellulose [10]. Besides genetic factors, husbandry systems also make important contributions to the diversity of cecum microflora. Compared with caged chickens, free-range chickens have free access to feed herbage, seeds and insects, leading to accumulation of many intestinal microbes from the environment and formation of cecum microbial communities. Therefore, studies on the formation and diversity (species and richness) of the cecum microflora are of great significance for improving animal production performance and product quality.

In recent years, cecum microorganism has been a research focus, and a range of techniques have been developed to facilitate its research, such as denaturing gradient gel electrophoresis [11], terminal restriction fragment length polymorphism (T-RFLP) [12], real-time quantitative reverse transcription-polymerase chain reaction (qRT-PCR) [13] and anaerobic cultivation [14]. In this study, Illumina MiSeq sequencing was adopted due to its advantages of high resolution for characterization of the microbial community, large coverage and low cost [15].

Diet has been reported as the biggest determinant of the composition of chicken intestinal flora [16]. The primary aim of this study was to explore the effects of the two variables (husbandry system and strain) on the chicken cecum microorganisms under the same dietary conditions. The results of this study can provide theoretical guidance to the farmers who are engaged in ecological breeding of indigenous chickens.

## MATERIALS AND METHODS

### Animal care

Animal experiments were performed under the institutional guidelines of Ecological Chicken Farm in Jianshi County of Hubei Province and Laboratory of Poultry Genetics, College of Animal Science and Veterinary Medicine, Huazhong Agricultural University, China. The experiments were performed according to recommendations proposed by the European Commission (1997) to minimize the suffering of animals.

### Experimental design and administration

Jingyang chickens were reared in the Fire Phoenix Ecological Breeding Cooperative of Jianshi County (Hubei, China). A total of 320 laying hens (body weight, 1.70±0.15 kg; 47 weeks old), derived from silver feather (S-line = 160 hens) and gold feather (G-line = 160 hens), under the two husbandry systems of enrichment cages (EC) and free range (FR) were randomly divided into four groups (80 hens per group): silver-feathered hens in enrichment cages (SEC), silver-feathered hens in free range (SFR), gold-feathered hens in enrichment cages (GEC), and gold-feathered hens in free range (GFR). For EC husbandry system, each hen was reared in an individual cage (70×60×75 cm) with controlled conditions of 18°C to 22°C, 16L/8D photoperiod/day. For FR husbandry system, chickens were raised in mountain areas characterized by arbor evergreen coniferous in the visible small stones and grains of sand, with a stocking density of 1.5 chickens per 10 square meters. Chickens in EC and FR were fed the same local standard diet (2,700 kcal/kg, 13% protein, 1% calcium, 0.45% phosphorous) (DB 42/T 686-2011) and drinking water *ad libitum* during the experiment period.

### Sampling and measurements

At the end of the 60-day experiment, 5 chickens were randomly selected from each treatment (a total of 4 treatments × 5 repeat = 20) and the cecal samples were collected in a cryopreservation tubes and then mixed and stored in liquid nitrogen at −70°C.

Microbial genome DNA was extracted from cecal samples by using QIAamp DNA stool mini kit (Tiangen Biotech, Beijing, China) according to the manufacturer’s instructions. PCR amplification of the V3–V4 region of the 16S ribosomal RNA (rRNA) gene was performed using the 341F/802R primer set (341F: CCTAYGGGRBGCASCAG; 802R: GGACTAC NNGGGTATCTAAT) as previously reported [17]. Only the products without primer dimers and contaminant bands were used for 16S rDNA high throughput sequencing at Shanghai Personal Biotechnology Co., Ltd (Shanghai, China).

### Data analysis

The screened and obtained high-quality sequences uploaded to quantitative insights into microbial ecology (QIIME, v1.8.0), were clustered into operational taxonomic units (OTUs) with a sequence similarity of >97% using the USEARCH software.

## RESULTS AND DISCUSSION

### Operational taxonomic unit analysis results of cecum microbes

**Figure 1:**
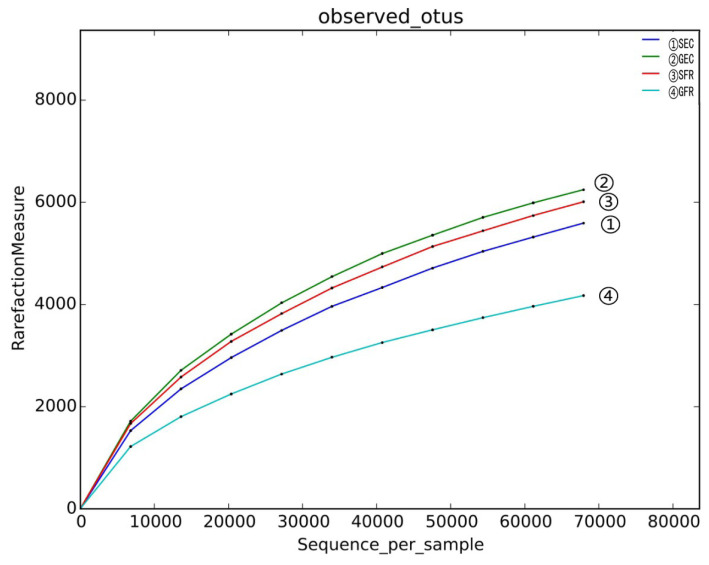
Species rarefaction curve. There is no intersection point after the zero point of the curve, and the order from high to low is GEC, SFR, SEC, GFR respectively.

The OTU analysis results of cecum microbes are presented in Table 1 and 2. A total of 22,660 OTUs consisted of 5,880, 6,245, 6,316, and 4,219 OTUs in SEC, GEC, SFR, and GFR, respectively (Table 1), and the annotation results are shown at various classification levels and the number of annotations in species is far less than that in genus level (Table 2). Moreover, the rarefaction curve (Figure 1) tends to be flat as sequencing depth increases and the number of OTUs reaches a plateau, with a sample coverage rate of over 0.96 (Table 3). These results confirmed that our sequencing has covered most of the sample species, and the sequencing results fully reflect the microbial diversity of the samples.

### Abundance and diversity analysis of cecum microbiota

The results of abundance and diversity analysis are presented in Table 3. In silver-feathered chickens, Shannon index and Simpson index of SFR were higher than those of SEC. Nevertheless, compared with GFR, GEC showed higher values of chao1, Shannon index, and Simpson index. Bailey et al [18] found that exposure to prolonged restraint pressure (a stress method used to induce physiological responses in an animal by restricting its free movement) results in a decrease in the abundance and diversity of intestinal flora in mice. Thus, some of the above results in the present study could be explained by a prolonged restraint pressure-induced decrease in the relative abundance of bacteria of chickens in cages. However, Chao1, a species richness index, was lower in free-range groups than in cage groups. In our former research, a higher level of lactobacilli was observed in free range group, which could inhibit the proliferation of other bacteria and promote gut health [19]. GFR showed the maximum content of Lactobacillus, but the minimum content of chao1, Shannon index, and Simpson index in our present study. These data demonstrated that Lactobacillus did inhibit the growth of some bacteria.

### Microbial community composition in cecum

The core microbiota of the 4 groups were counted at the phylum level (Table 4) and genus level (Table 5). At the phylum level, core members are microbes belonging to Bacteroidetes (49% to 60%), Firmicutes (21% to 32%), and Proteobacteria (2% to 4%), which are similar to the results reported in broilers [20], Dagu chickens [21], and Tibetan Chickens [22]. It is reported that the total proportion of Firmicutes and Bacteroides is even more than 90% in some mammalian species [23,24], and the Firmicutes/Bacteroides ratio can directly reflect the capacity of energy absorption and storage of host, such as the increase of the proportion of Firmicutes due to the deposition of fat [25], or the increase of the proportion of Bacteroides with the decrease of the Firmicutes/Bacteroides ratio due to enormous absorption of cellulose food [23,26]. In our study, the total proportion of Bacteroidetes and Firmicutes was more than 75% in Jingyang chickens, which was higher in free-range groups (SFR 81.61%; GFR 82.12%) than in enrichment-cage groups (SEC 76.38%; GEC 75.33%). The ratio of Firmicutes/Bacteroides was lower in GFR (23.00%/59.12%) than in GEC (21.20%/54.13%), whereas opposite results were obtained for silver-feather hens (SFR vs SEC). The reduced ratio of Firmicutes/Bacteroides in GFR group may be attributed to the higher cellulose food intakes in free range environment.

The core bacteria were Bacteroides (26% to 31%), Rikenellaceae (9% to 16%), Parabacteroides (2% to 5%), and Lachnoclostridium (2% to 6%) at the genus level. Ruminococcaceae was higher in SFR (2.25%) than in SEC (1.23%) as well as higher in GFR (1.4%) than in GEC (0.78%). Combined with our previous research results on cecum index, we speculate that the increased of free-range husbandry in the accumulation of microorganisms is related to cellulose degradation, which can be supported by the studies of Saengkerdsub et al [27], Lu et al [28], and Latham et al [29], who reported that ruminococcaceae was responsible for cellulose digestion.

### Abundance and clustering analysis of relative bacterial communities

**Figure 2:**
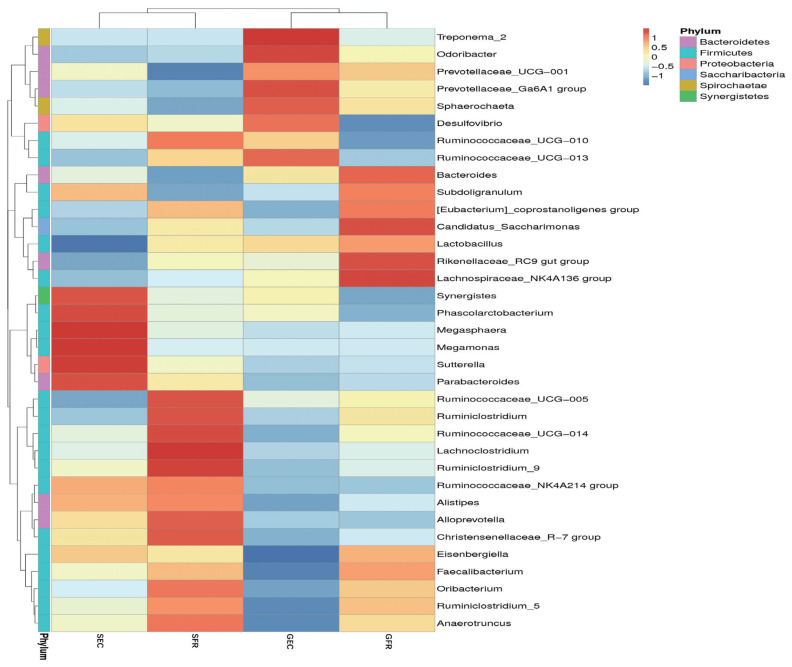
Heat maps of species abundance and clustering. The phylum classification position clustering (horizontal) and top 35 phylum sample clustering (vertical clustering). Different color means the different relative abundance of the phylum in all the four samples.

A further analysis was conducted to discover the relative bacterial community abundance at the phylum level, and the results are shown in Figure 2. The phyla were divided into four groups. Group 1 (Synergistes, Phascolarctobacterium, Megasphaera, Megamonas, Sutterella, and Parabacteroides) was predominant in SEC. Group 2 (Ruminococcaceae, Lachnoclostridium, Alloprevotella, Christensenellaceae, Oribacterium, and Anaerotruncus) was typical in SFR. Group 3 (Treponema, Odoribacter, Prevotellaceae, Sphaerochaeta, Desulfovibrio, and Ruminococceae) and Group 4 (Bacteroides, Subdoligranulum, Eubacterium, Saccharimonas, Rikenellaceae, and Lachnoclostridium) were observed in GEC and GFR, respectively. The clustering was dependent on hen strains (gold vs silver).

The clustering result was consistent with the strain classification: the silver-feather strain and the gold-feather strain. Actually, factors affecting the intestinal microbiotas are host and environment, with the former mainly referring to heredity, age and endocrinology, and the latter involving diet, geographical environment, lifestyle, and the use of antibiotics [16,30]. Diet has been reported as the dominant factor for the intestinal microbial community in the formation of host genotype [16]. In this study, we explored other factors affecting cecum microbial diversity under the same dietary conditions. Our study proved that the genetic factor has a greater impact than husbandry system on cecum microbial diversity in Jingyang chickens.

## CONCLUSION

In conclusion, Bacteroides is the most abundant in cecum microbiotas of Jingyang chickens, followed by Firmicutes and Proteobacteria. Free range husbandry system as well as strain have observable effects on diversity of the cecum microorganism of Jingyang chickens. However, genetic factors show a more significant influence than husbandry system on cecum microbial diversity of chickens when fed the same diet.

## Figures and Tables

**Table 1:** Sequencing data and OTUs annotation

Group1)	Raw PE2)	Raw tags2)	Clean tags2)	Effective tags2)	Avg Len2)	OTUs number2)
SEC	100,563	86,795	83,500	81,123	456	5,880
SFR	102,343	89,821	86,752	83,586	455	6,316
GEC	92,222	79,597	76,626	73,960	457	6,245
GFR	86,700	75,441	72,728	72,027	457	4,219

OTU, operational taxonomic units.

1) SEC, silver-feathered hens in enrichment cages; SFR, silver-feathered hens in free range; GEC, gold-feathered hens in enrichment cages; GFR, gold-feathered hens in free range.

2) Raw PE, the original PE (paired-end) reads; Raw tags, tag sequences obtained after splicing; Clean tags, a sequence with low-quality and short-length tags being removed; Effective tags, tag sequence collected for further analysis after filtering the chimera; Avg Len, the average length of effective tags; OTUs number, the number of OTUs obtained per sample.

**Table 2:** Classification of OTUs annotation results

Group1)	Unassigned	Kingdom	Phylum	Class	Order	Family	Genus	Species
SEC	1,353	1	20	5	92	719	3,673	17
SFR	1,332	4	19	4	87	733	4,119	18
GEC	1,443	2	17	4	101	721	3,944	13
GFR	727	2	12	4	71	541	2,855	7

OTU, operational taxonomic units.

1) SEC, silver-feathered hens in enrichment cages; SFR, silver-feathered hens in free range; GEC, gold-feathered hens in enrichment cages; GFR, gold-feathered hens in free range.

**Table 3:** Abundance and diversity analysis of cecum microbiota

Group1)	Chao1	Shannon	Simpson	observed species	Goods coverage
SEC	8367.58	9.05	0.99	5591.1	0.96
SFR	8218.05	9.32	0.991	6009.3	0.96
GEC	8028.44	9.13	0.989	6244.9	0.97
GFR	6752.82	8.44	0.987	4173.7	0.97

1) SEC, silver-feathered hens in enrichment cages; SFR, silver-feathered hens in free range; GEC, gold-feathered hens in enrichment cages; GFR, gold-feathered hens in free range.

**Table 4:** Phylum distribution of cecum bacteria

Items	SEC1)	SFR1)	GEC1)	GFR1)
Bacteroidetes	50.12	49.66	54.13	59.12
Firmicutes	26.26	31.95	21.2	23
Proteobacteria	3.26	2.87	3.11	2.35
Spirochaetae	0.82	0.78	2.21	1.04
Synergistetes	1.31	0.59	0.78	0.23
Saccharibacteria	0.3	0.38	0.26	0.71
SHA-109	0.54	0.6	0.17	0.23
Actinobacteria	0.53	0.6	0.21	0.59
Verrucomicrobia	0.21	0.29	0.05	0.12
Euryarchaeota	0.15	0.12	0.09	0.21
Others	16.51	12.15	17.81	12.39

1) SEC, silver-feathered hens in enrichment cages; SFR, silver-feathered hens in free range; GEC, gold-feathered hens in enrichment cages; GFR, gold-feathered hens in free range.

**Table 5:** Genus distribution of cecum bacteria

Items	SEC1)	SFR1)	GEC1)	GFR1)
Bacteroides	27.87	26.42	28.86	30.55
Rikenellaceae	9.03	11.73	11.29	15.31
Parabacteroides	4.75	3.45	2.24	2.52
Lachnoclostridium	3.37	5.1	2.75	3.51
Desulfovibrio	2.32	2.21	2.56	1.89
Prevotellaceae	2.35	1.61	4.03	3.15
Ruminococcaceae	1.23	2.25	0.78	1.4
Christensenellaceae	1.11	1.52	0.6	0.8
Others	47.98	45.71	46.89	40.86

1) SEC, silver-feathered hens in enrichment cages; SFR, silver-feathered hens in free range; GEC, gold-feathered hens in enrichment cages; GFR, gold-feathered hens in free range.
